# The roles of microRNAs in the regulation of tumor metastasis

**DOI:** 10.1186/s13578-015-0028-8

**Published:** 2015-06-20

**Authors:** Lei Zhou, Fan Liu, Xiaomin Wang, Gaoliang Ouyang

**Affiliations:** Fujian Provincial Key Laboratory of Chronic Liver Disease and Hepatocellular Carcinoma, Zhongshan Hospital, Medical College, Xiamen University, Xiamen, 361004 China; Department of Cardiology, The First Affiliated Hospital of Nanjing Medical University, Nanjing, 210029 China; Medical College, Xiamen University, Xiamen, 361102 China; State Key Laboratory of Cellular Stress Biology, Innovation Center for Cell Signaling Network, School of Life Sciences, Xiamen University, Xiamen, 361102 China

**Keywords:** microRNA, Cancer, Metastasis, Epithelial-mesenchymal transition, Cancer stem cell

## Abstract

MicroRNAs (miRNAs) are small noncoding regulatory RNAs that regulate gene expression post-transcriptionally by either inhibiting protein translation or degrading target mRNAs. The differential expression profiles of miRNAs in different types of cancers and in the multi-step process of tumor progression indicate that miRNAs are involved in tumor onset, growth and progression. Metastasis is the most common cause of cancer-related mortality. Current evidence demonstrates that aberrant miRNA expression promotes or inhibits tumor metastasis by modulating the expression of numerous target genes. Therefore, the identification of metastasis-related miRNAs and a better understanding of the complex functions of miRNAs in tumor metastasis will provide potential diagnostic and prognostic biomarkers, as well as therapeutic targets for clinical application. Here, we review the functions of miRNAs in the control of multiple steps of tumor metastasis.

## Introduction

Metastasis remains a major challenge for the clinical management and prognosis of patients with cancer. As the most common cause of cancer-related mortality, metastasis involves several continuous steps through which cancer cells disseminate and spread from a primary tumor to distant sites, forming secondary tumors in other organs and tissues [1–3]. Recent studies have provided exciting new insights into the molecular mechanisms of tumor metastasis; however, research on the functions and mechanisms of microRNAs (miRNAs) in metastasis has only recently begun.

miRNAs are highly conserved, endogenous noncoding RNAs that have been shown to regulate distinct cellular processes by interfering with protein expression at the post-translational level. Specifically, miRNAs modulate gene expression by binding to the 3’-untranslated-region (UTR) of target mRNAs, which have complete or partial complementarity with their seed region. This results in impairment of the translation and/or stability of the target mRNAs and the subsequent downregulation of protein expression. Current evidence has demonstrated that many miRNAs that have normal functions in the modulation of physiological processes are deregulated in various pathological processes, including tumor onset, growth and metastasis [4–6]. A single miRNA can function as an oncogene or as a tumor suppressor gene to silence the expression of many target genes and thereby remodel the expression profile of cells. Thus, specific miRNAs can function as promoters or suppressors of metastatic progression and coordinately inhibit numerous target genes in tumor metastasis. Aberrant miRNA expression profile during multi-step metastatic processes is a common characteristic of tumor metastasis [7, 8]. It was recently discovered that secreted miRNAs have also been found in the serum and other body fluids of individuals with cancer [9, 10]. Acting in a manner consistent with their primary roles, secretory miRNAs also act as translational inhibitors in recipient cells, which indicates that secretory miRNAs can regulate cellular processes at distant sites during the tumor metastatic cascade. Therefore, miRNAs may be good targets for the modulation of multiple steps of the metastatic cascade. In this review, we focus on the roles of miRNAs in the regulation of tumor metastasis, and we discuss the possibility of miRNAs as diagnostic and prognostic biomarkers and therapeutic targets for tumor metastasis.

### Functions of miRNAs in the tumor metastatic cascade

#### miRNAs and local invasion

Some tumors rarely disseminate to distant target sites although they are locally invasive, whereas other tumors are highly aggressive and establish metastatic lesions at secondary sites at a high frequency. To migrate to secondary sites and to flourish in those sites, disseminated tumor cells (DTCs) that have detached from the primary tumor microenvironment must survive a series of steps termed the metastatic cascade. Cancer cell detachment, migration and invasion are early steps in the invasion and metastatic cascade. Current evidence demonstrates that miRNAs play critical roles in tumor cell invasion. miR-10b, which promotes cell migration and invasion, has been identified as a metastasis-promoting miRNA in breast cancer [11]. The silencing of miR-10b reduces mesenchymal subtype-like glioma cell invasion through the suppression of TP53, PAX6, NOTCH1 and HOXD10 [12]. The miR-17-92 cluster is often amplified in cancers. The miR-17-92 cluster member miR-19a/b is highly expressed in gastric cancer tissues and is significantly associated with gastric cancer metastasis. miR-19a/b promotes the migration, invasion and metastasis of gastric cancer cells by targeting MXD1, an antagonist of c-Myc [13]. miR-135b expression is also increased in invasive non-small-cell lung cancer cells. miR-135b promotes cell migration and invasion *in vitro* and increases cancer metastasis *in vivo* via the regulation of the Hippo pathway [14]. miR-362-5p is highly expressed in hepatocellular carcinomas (HCCs) and correlates with HCC progression. The inhibition of miR-362-5p in HCC cells suppresses cell proliferation, migration and invasion *in vitro* and tumor growth and metastasis *in vivo* [15].

In contrast, some miRNAs are negative regulators of cell invasion. miR-34a and miR-34c are significantly downregulated in metastatic breast cancer. The restoration of miR-34a/c in breast cancer cell lines inhibits cell migration and invasion *in vitro* and reduces distal pulmonary metastasis *in vivo* by directly targeting Fra-1 [16]. Androgen-regulated miR-135a inhibits prostate the migration and invasion of cancer cells directly through its downstream targets, ROCK1 and ROCK2 [17]. HMGA1 activates miR-137 transcription by binding to the promoter of miR-137 in colorectal cancer cells, which reduces the level of FMNL2, a downstream target of miR-137. The ectopic expression of miR-137 reduces the invasiveness of colorectal cancer cells [18]. miR-145 attenuates gastric cancer cell migratory and invasive abilities *in vitro* by targeting N-cadherin (CDH2). Assays to detect experimental and spontaneous metastasis further confirmed that miR-145 suppresses the metastatic cascade *in vivo* [19]. The overexpression of miR-145 inhibits the invasiveness and metastasis of neuroblastoma cells by targeting HIF-2α [20]. miR-1 expression is consistently downregulated in primary human prostate tumors and is reduced even more in distant metastases. As a prostate cancer suppressor, miR-1 affects the cellular organization of F-actin and impairs tumor cell invasion and the formation of filopodia [21]. These findings indicate that miRNAs play critical roles in the regulation of local invasion by cancer cells.

The breakdown and remodeling of the extracellular matrix are critical steps in cancer cell invasion. Tenascin C and other matricellular proteins, such as periostin and osteopontin, play important roles in remodeling the tumor metastatic microenvironment [22, 23]. The loss of miR-335 expression is related to poor distal metastasis-free survival of patients with breast cancer. The restoration of miR-335 expression suppresses cell migration, invasion and metastasis by targeting tenascin C and SOX4 in breast cancer [24]. miR-29c, which is downregulated in nasopharyngeal carcinomas, targets several genes that encode extracellular matrix proteins, including multiple collagens and laminin γ1; these proteins are associated with increased tumor invasion and metastasis [25]. Therefore, miRNAs are believed to be coordinated regulators of the remodeling of the extracellular matrix during cancer cell invasion.

Many cancer cells undergo epithelial-mesenchymal transition (EMT) to achieve enhanced motility and to gain resistance to apoptosis; however, some cancer cells might undergo collective migration independent of an EMT program. The miR-34/SNAIL and miR-200/ZEB mutual-inhibition feedback circuits contribute to the regulation of epithelial-hybrid-mesenchymal fate determination and collective migration [26]. miR-21 is involved in epithelial collective cell migration [27]. Recently, it was shown that miR-124 directly regulates the stability and translation of integrin β1 mRNA in order to modulate the intercellular adhesion of the leading cells in tumors during collective invasion [28]. However, little is known at present regarding the role of miRNAs in the regulation of collective migration in the tumor metastatic cascade.

#### miRNAs and intravasation

To disseminate to distant sites, invasive cancer cells must enter the circulatory or lymphatic systems or travel across the body cavities. The destruction of vascular endothelial barriers is a critical step for cancer cell intravasation. miR-105 is secreted by metastatic breast cancer cells and promotes metastasis by direct targeting of the tight junction protein ZO-1, which destroys vascular endothelial barriers [29]. Compared with normal breast tissues, miR-21 is highly expressed in breast tumors and correlates with advanced stage, lymph node metastasis and reduced survival time. The suppression of miR-21 significantly reduces the invasion and lung metastasis of breast cancer cells by targeting TPM1, PDCD4 and Maspin [30]. In addition to the promotion of cell invasion and proliferation, miR-21 can enhance colorectal cancer cell intravasation by binding to the 3′-UTR of PDCD4. The downregulation of miR-21 in colorectal cancer cells inhibits invasion, intravasation and lung metastasis [31]. miR-182 is significantly overexpressed in mouse sarcomas that metastasize to the lungs. miR-182 deletion decreases circulating tumor cells, whereas miR-182 overexpression increases circulating tumor cells, which indicates that miR-182 promotes the entry of cancer cells into the circulation [32].

The expression of members of the miR-520c/miR-373 family negatively correlates with lymph node metastasis, specifically in ER-negative breast cancers. The miR-520c/miR-373 family inhibits cell invasion *in vitro* as well as the intravasation of breast cancer cells *in vivo* and plays a tumor-suppressive role in ER-negative breast cancers via the regulation of the NF-κB and TGF-β pathways [33]. RKIP inhibits breast cancer cell intravasation and bone metastasis in mice in part through a signaling cascade that involves MAPK, Myc, LIN28, let-7 and downstream let-7 targets, which indicates that let-7 may be involved in RKIP-inhibited cell intravasation [34]. These findings have opened new avenues in our understanding of the roles of miRNAs in the regulation of cancer cell intravasation during tumor metastasis.

#### miRNAs and the systemic circulation

Anoikis is a special form of programmed cell death that occurs when normal cells detach from the extracellular matrix or from cell–cell anchors and are maintained in suspension. Under normal circumstances, anoikis prevents the attachment of these detached cells to an inappropriate extracellular matrix and prevents the colonization of these cells outside of their usual anatomical site. After they enter the circulation, some cancer cells are destroyed in the bloodstream by shear stress and by anoikis. Therefore, DTCs must still overcome detachment, evade immune attack and survive the sheer forces that they encounter in the circulation. Several lines of evidence have indicated that p53 and various other proteins play important roles in anoikis, which is triggered by a lack of adhesion; however, the specific miRNAs that regulate anoikis in the systemic circulation are unknown.

The level of miR-296-3p is much higher in highly metastatic human prostate cancer cells than in non-metastatic cells. The knockdown of miR-296-3p in metastatic human prostate cancer cells reduces their resistance to natural killer (NK) cells, whereas the ectopic overexpression of miR-296-3p in non-metastatic prostate cancer cells enhances the tolerance of these cells to NK cells. miR-296-3p promotes prostate cancer metastasis through an augmentation of the survival of NK cell-resistant circulating tumor cells by targeting ICAM-1 [35]. miR-148b is downregulated in aggressive breast tumors and inhibits multiple steps of tumor progression via the regulation of invasion, resistance to anoikis, extravasation, metastatic colonization in lung and chemotherapeutic response [36]. Although several miRNAs contribute to cell survival and help cells to overcome the shear stress and immune attack, little is known in regards to the molecular mechanisms of miRNAs in the systemic circulation of cancer cells.

#### miRNAs and extravasation

For blood vessel-borne metastasis, cancer cells must arrest or adhere to the endothelial lining and then undergo extravasation. As a potent regulator of ZO-1, miR-105 can be expressed and secreted by metastatic breast cancer cells, which can then disrupt the tight junctions of endothelial cells. In endothelial monolayers, exosomal miR-105, which is secreted by breast cancer cells, not only destroys the endothelial barriers in primary sites but also disrupts the tight junctions of endothelial cells in secondary organs, which allows cells to extravasate. miR-105 overexpression in non-metastatic cancer cells promotes vascular permeability and metastasis to distant sites, whereas miR-105 inhibition in highly metastatic tumors alleviates these effects [29]. Thus, miR-105 can efficiently destroy the integrity of vascular endothelial barriers to promote metastasis. miR-155 is frequently highly expressed in several types of human cancers. miR-155-expressing breast tumors are associated with an aggressive malignant phenotype and with poor prognosis [37]. Interestingly, the loss of miR-155 reduced central nervous system extravasation of systemic tracers in mice. Moreover, miR-155 negatively regulates the function of the brain endothelial barrier by through the targeting of the cell-cell complex molecules annexin-2 and claudin-1 and the focal adhesion components DOCK-1 and syntenin-1 [38], which indicates that miR-155 may promote cancer cell extravasation during the process of tumor metastasis. miR-214 expression is deregulated in several human tumors. It suppresses cell migration and invasion *in vitro* and inhibits the metastasis of colorectal cancer to the liver *in vivo* by targeting FGFR1 [39]. In contrast, miR-214 promotes melanoma metastasis through an enhancement of extravasation [40]. miR-214 overexpression in melanoma cells not only promotes cell migration, invasion and resistance to anoikis *in vitro* but also promotes extravasation and the formation of lung metastasis *in vivo* [41].

The expression of miR-31 expression is inversely correlated with metastasis in patients with breast cancer. miR-31 represses several metastatic processes, including local invasion, extravasation, initial survival in distant tissues and distant colony formation. The shRNA-mediated concurrent suppression of ITGA5, RDX and RhoA is sufficient to phenocopy the influences of miR-31 on local invasion, early post-intravasation events and metastatic colonization *in vivo* [42]. A recent report demonstrates that overexpression of miR-25 in highly metastatic human prostate cancer cells attenuates metastasis via the inhibition of extravasation *in vivo* by direct targeting of αv- and α6-integrins [43]. These findings suggest that miRNAs can regulate tumor metastasis through the modulation of extravasation.

#### miRNAs and metastatic colonization

Metastatic colonization is the key rate-limiting step in the tumor metastasis cascade. The overexpression of miR-200 s correlates with an increased risk of metastasis in breast cancer and augments metastatic colonization in mice partly through the direct targeting of Sec23a [44]. Breast cancer patients with metastasis have a high level of circulating miR-122. Breast cancer-cell-secreted miR-122 promotes metastatic colonization in the brain and lungs of mice and inhibits glucose uptake by niche cells through the targeting of pyruvate kinase. This process results in an increase in glucose that is available to the cancer cells that are undergoing metastasis in pre-metastatic niches [45].

Profile analyses have revealed that the level of miR-149 is extremely low in basal breast cancer. The expression of miR-149 significantly inhibits basal-like breast cancer cell migration and invasion *in vitro* and impairs lung colonization *in vivo* [46]. For successful metastatic colonization in the liver, DTCs must settle and adapt to the unfavorable microenvironment of the liver parenchyma and resume proliferation. The former metastatic steps are relatively easy to overcome because of direct blood circulation via portal veins and because of the porous property of the liver sinusoids. miR-493 inhibits the settlement of colon cancer cells that have metastasized in the liver parenchyma and promotes the death of these cells by targeting IGF1R [47]. miR-148a is abundant in the liver but is dramatically downregulated in HCC tissues. The restoration of miR-148a expression in hepatoma cells markedly inhibits their migration *in vitro* and their pulmonary metastatic colonization *in vivo* [48]. Collectively, these findings demonstrate that many miRNAs are involved in the regulation of metastatic colonization.

### Functions of miRNAs in EMT during tumor metastasis

EMT is the critical program that is used by DTCs for invasion and metastasis. In patients with breast cancer, the miR-9 levels in primary tumors from patients with diagnosed metastases are markedly upregulated compared with those from metastasis-free patients. miR-9 can promote the motility and invasiveness of breast cancer cells by targeting CDH1, the E-cadherin-encoding messenger RNA [49]. A high level of miR-155 is frequently observed in invasive breast cancer tissues. miR-155 knockdown inhibits TGF-β-induced EMT, dissolution of tight junction, cell migration and invasion [50]. Patients with gallbladder carcinoma with higher miR-20a levels exhibit worse overall survival than those with lower miR-20a levels. miR-20a is closely correlated with local invasion, distant metastasis, and poor prognosis in patients with gallbladder carcinoma. The overexpression of miR-20a in gallbladder carcinoma cells induces EMT and promotes metastasis via the direct inhibition of Smad7 [51].

miR-200 family members are important markers of epithelial cells and are critical master regulators of EMT. Under normal conditions, miR-200 can inhibit the expression of ZEB1 and ZEB2, which promotes an epithelial phenotype and cell adhesion. However, ZEB1 and ZEB2 can be activated by the contextual signals within the tumor microenvironment and can suppress the expression of their own suppressor, miR-200, to promote EMT, invasion and metastasis [52, 53]. The level of miR-203 is extremely low in highly metastatic breast cancer cells, and the overexpression of miR-203 in these cells suppresses cell invasion *in vitro* and metastatic colonization of the lung *in vivo* via the targeting of SNAI2. Moreover, miR-203 and SNAI2 inhibit the expression of each other through a negative feedback loop, which modulates EMT and metastasis [54]. miR-124 is frequently downregulated in HCC tissues and is negatively correlated with a poor prognostic phenotype in patients with HCC. The overexpression of miR-124 in HCC cells inhibits EMT and the formation of stress fibers (i.e., filopodia and lamellipodia) *in vitro* and suppresses intrahepatic and pulmonary metastasis *in vivo*, most likely by targeting ROCK2 and EZH2 [55]. The expression of miR-612 in patients with HCC is inversely correlated with tumor size, stage, EMT and metastasis. miR-612 contributes to both the initial and final steps of the metastatic cascade by inhibiting local invasion and distant metastatic colonization [56]. miR-125a is a recently identified regulator of myeloid malignancies that targets WT1. miR-125a knockout mice develop myeloproliferative disorders and exhibit urogenital abnormalities [57]. Moreover, miR-125a can inhibit EMT in ovarian cancer cells by targeting ARID3B [58]. Therefore, miRNAs are critical coordinating regulators of EMT during tumor metastasis.

Only a small subset of DTCs has the potential to initiate metastatic growth in a new tissue environment [22]. Once they become established in a new tissue environment, metastatic cancer cells revert to a non-metastatic epithelial phenotype via mesenchymal-epithelial transition (MET). Twist1 can induce EMT to facilitate breast cancer cell intravasation into the circulation and the subsequent dissemination of these cells into the lungs. However, for DTCs to proliferate and form metastases at distant sites, Twist1 must be turned off to allow MET to proceed [59]. Interestingly, Twist1 can promote the expression of miR-10b by directly binding to the promoter of miR-10b [11], which indicates that miR-10b may affect the invasion, intravasation, circulation, extravasation and metastatic colonization of breast cancer cells. Recently, miR-424 has been shown to be positively associated with EMT-like phenotypes and with Twist1/2 expression. miR-424 is highly expressed in primary breast tumors compared with matched normal breast tissues; however, miR-424 expression is decreased in metastases compared with matched primary breast tumors [60]. These findings demonstrate that EMT and/or MET are critical programs used by cancer cells for metastasis.

### Functions of miRNAs with respect to cancer stem cells in tumor metastasis

Cancer stem cells (CSCs) are a sub-population of cancer cells that are thought to be responsible for tumor initiation, metastasis, resistance to therapy and relapse [22, 61, 62]. Aberrant expression of miRNAs may play a critical role in the regulation of the characteristics and functions of CSCs in tumor metastasis. The impairment of the miRNA biogenesis gene DICER1 in colorectal cancer cells promotes tumor initiation and metastasis by endowing colorectal cancer cells with stem cell properties and EMT phenotypes [63]. miR-22 overexpression is associated with poor clinical outcomes of breast cancer patients and promotes EMT, invasiveness, stemness and metastasis [64]. A high level of miR-199a correlates with poor survival in patients with breast cancer. Mesenchymal stem/stromal cells promote the aberrant expression of miR-199a in breast cancer cells in a contact-dependent manner. Subsequently, miR-199a inhibits the expression of FOXP2 and endows breast cancer cells with enhanced CSC properties [65]. miR-9 is highly expressed in metastatic human primary squamous cell carcinoma. A high level of miR-9 enhances the expansion and metastasis of squamous cell carcinoma CSCs [66]. Increased miR-133a is significantly correlated with poor prognosis in patients with osteosarcoma. The silencing of miR-133a by locked nucleic acids (LNAs) inhibits the invasiveness of CSCs that express high levels of CD133. The administration of LNAs with chemotherapy synergistically inhibits lung metastasis and prolongs the survival of osteosarcoma-bearing mice [67].

As a key negative regulator of prostate CSCs, miR-34a suppresses prostate CSCs and metastasis by directly targeting CD44 [68]. miR-7 is downregulated in breast CSCs. The overexpression of miR-7 can suppress cell invasion and metastasis through a decrease in the breast CSC population and by a partial reversal of the EMT phenotype in breast cancer cells by targeting SETDB1 [69]. Interestingly, by targeting KLF4, miR-7 overexpression inhibits the ability of breast CSCs to metastasize to the brain [70]. We recently demonstrated that the expression level of miR-33b is extremely low in human breast cancer samples and in highly metastatic breast cancer cell lines. Moreover, miR-33b can reduce the stemness and inhibit the metastasis of breast cancer cells to the lungs through the regulation of HMGA2, SALL4 and Twist1 [71]. Therefore, miRNAs are critical players in the modulation of the stemness of DTCs in the metastatic cascade.

### Functions of miRNAs in the metastatic microenvironment

DTCs at distant sites must evade immune attack or recruit immune/inflammatory cells or other stromal cells and their secreted factors to assist them so that they can adapt and remodel the metastatic microenvironment and complete the subsequent proliferation and colonization steps of the metastatic cascade [2, 10].

Portal vein tumor thrombus (PVTT) is highly related to poor prognosis in individuals with HCC. miR-34a can decrease the production of the chemokine CCL22, which recruits regulatory T cells to facilitate immune escape. Moreover, miR-34a expression can be inhibited by TGF-β. Interestingly, HBV infection and the activity of the TGF-β-miR-34a-CCL22 axis predispose HCC patients to the development of PVTT, possibly through the formation of an immune-supportive microenvironment that favors the colonization of disseminated HCC cells in the portal venous system [72]. Recently, miR-34a was identified as a novel suppressor of osteoclastogenesis, bone resorption and the bone metastatic niche. The metastasis of breast and skin cancers to bone is diminished in osteoclastic miR-34a transgenic mice but is exacerbated in miR-34a knockout and heterozygous mice. In addition, miR-34a nanoparticle treatment can attenuate the metastasis of breast and skin cancers to bone. Moreover, miR-34a is downregulated during osteoclast differentiation and is a newly identified suppressor of osteoclastogenesis and bone resorption. Therefore, osteoclastic miR-34a can effectively inhibit malignant progression by disturbing the bone metastatic niche [73]. In addition, the levels of two other osteoclast miRNAs, miR-16 and miR-378, are increased in bone lesions and in serum samples from patients with bone metastasis and correlate with the bone metastasis burden [74]. Thus, these osteoclastic miRNAs are potential biomarkers and therapeutic targets of bone metastasis.

miR-155 is involved in the development and function of immune cells [75]. The knockdown of miR-155 in the myeloid compartment of MMTV-PyMT mice significantly promotes tumor growth by impairing the activation of tumor-associated macrophages [76]. Bone marrow that lacks miR-155 significantly promotes lung metastasis but does not have a substantial effect on primary tumor growth. The expression of miR-155 in host immune cells inhibits solid tumor metastasis by regulating the recruitment and polarization of bone marrow-derived macrophages [77]. miR-155 deficiency promotes the growth of melanoma and Lewis lung carcinoma tumors by enhancing the recruitment of myeloid-derived suppressor cells (MDSCs) to the tumor microenvironment [78]. However, a high level of miR-155 in leukemia cells correlates with aggressive disease in patients with chronic lymphocytic leukemia (CLL). miR-155 enhances cell sensitivity to B-cell receptor ligation, which indicates that miR-155 is involved in the regulation of the tumor microenvironment of CLL [79]. Recently, the inhibition of miR-155 by antisense oligomers was used for cancer therapy that was targeted to the tumor microenvironment in a mouse model of lymphoma [80]. MDSCs potently inhibit anti-tumor immune responses and contribute to the formation of a metastatic microenvironment that favors tumor metastasis. The expression of miR-494 promotes the accumulation of MDSCs in tumor tissues. The knockdown of miR-494 reverses the function of MDSCs in tumors and suppresses tumor growth and breast cancer cell metastasis *in vivo* [81]. MDSCs induce the expression of miR-101 in ovarian cancer cells, which subsequently augments the stemness of cancer cells and enhances their tumorigenic and metastatic potential [82].

Enhanced endothelial recruitment capacity is also a key feature of metastatic breast cancer cell populations. The silencing of miR-126 in breast cancer cells significantly promotes metastatic colonization in multiple organs, such as the lung, brain, bone and liver, via the enhancement of endothelial recruitment. Endogenous miR-126 can inhibit endothelial recruitment, metastatic angiogenesis and metastatic colonization of distant sites by coordinately targeting IGFBP2, PITPNC1 and MERTK [83]. Interestingly, miR-126 and miR-126* target SDF-1α to suppress breast cancer metastasis through the inhibition of the recruitment of mesenchymal stem cells and inflammatory monocytes [84]. Therefore, the recruitment of endothelial cells, mesenchymal stem cells and inflammatory monocytes, which is regulated by miR-126 and/or miR-126*, may provide incipient metastases with a critical microenvironmental cue that promotes the efficiency of metastatic initiation and results in enhanced metastatic colonization.

DTCs exist in a dormant state when they are in a hostile metastatic microenvironment but may switch from dormancy into proliferative metastatic growth in a supportive microenvironment. Tumor dormancy in metastatic sites can be regulated by intracellular signaling pathways and by extracellular microenvironmental cues [85, 86]. The exosomal transfer of miR-23b from bone marrow mesenchymal stem cells can promote breast cancer cell dormancy in a metastatic niche, as miR-23b targets MARCKS [87]. The CXCL12-specific miRNAs, including miR-127, −197, −222 and −223, which are transported from bone marrow-derived stromal cells to breast cancer cells via gap junctions or stromal-derived exosomes, promote breast cancer cell quiescence by a decrease in CXCL12 levels. This finding indicates that the transfer of miRNAs from bone marrow stroma to breast cancer cells can regulate the dormancy of metastatic breast cancer cells in the bone marrow [88]. However, to date, few miRNAs that maintain the dormant or active state of DTCs have been identified.

### miRNAs as diagnostic and prognostic biomarkers and therapeutic targets for tumor metastasis

miRNAs that are strongly associated with metastatic phenotypes may be useful as diagnostic or prognostic markers of metastasis. The overexpression of miR-103 and miR-107 in a mouse model of colorectal cancer was shown to promote local invasion and liver metastasis by targeting DAPK and KLF4. The signature of a high miR-103/107, low DAPK and low KLF4 expression profile correlates with the extent of lymph node and distant metastasis in patients with colorectal cancer. Thus, miR-103 and miR-107 can be used as prognostic markers for metastatic recurrence and for poor survival in patients with colorectal cancer [89]. The expression of miR-340 is significantly decreased in bone marrow cells of colorectal cancer patients with liver metastasis. Thus, miR-340 may be a novel prognostic factor and a therapeutic target for colorectal cancer [90]. The overexpression of miR-630 in breast cancer cells that are resistant to HER-targeted drugs significantly restores the efficacy of those drugs (e.g., lapatinib, neratinib and afatinib), which indicates that miR-630 can be used as a diagnostic and predictive marker to gauge the treatment response to HER-targeted drugs [91]. Further research in regards to miRNAs will facilitate the accurate and early identification of DTCs, will provide tractable targets for the monitoring of minimal residual disease and will help predict responses to treatment that is aimed at the prevention of metastasis.

The overexpression and inhibition of miRNA using miRNA mimics and inhibitors, respectively, might be useful for the treatment of tumor metastasis. miR-1908, miR-199a-5p and miR-199a-3p promote invasion, angiogenesis and colonization in the case of melanoma by the convergent targeting of ApoE. The therapeutic delivery of locked nucleic acids to silence these aberrantly expressed miRNAs significantly inhibits the metastasis of melanoma to multiple organs. Therefore, multiple prognostic miRNAs with synergistic combinatorial therapeutic potential can be used as a treatment for melanoma [92]. Many patients carry a large number of DTCs in their circulation, bone marrow and other distant sites at the time of their initial cancer diagnosis. Therefore, effective anti-metastatic treatment must inhibit the survival and proliferation of metastases that are already established. The induction of miR-31 in already-established metastases triggers pulmonary metastatic regression and extends survival, whereas the acute activation of miR-31 does not affect primary mammary tumor growth. This finding indicates that the restoration of miR-31 function may be used to specifically target metastatic disease [93]. The plasma level of miR-101 is higher in HCC patients without distant metastases compared with those with distant metastases. In an animal model of HCC, the delivery of lentivirus-mediated miR-101 inhibits HCC growth, intrahepatic metastasis and distant metastasis [94]. The administration of miR-10b antagomirs in mice with highly metastatic cells significantly inhibits lung metastases but does not affect primary mammary tumor growth [95]. Thus, miRNA mimics and inhibitors are potential candidates for the targeting of tumor metastasis.

## Conclusions and future perspectives

**Fig. 1:**
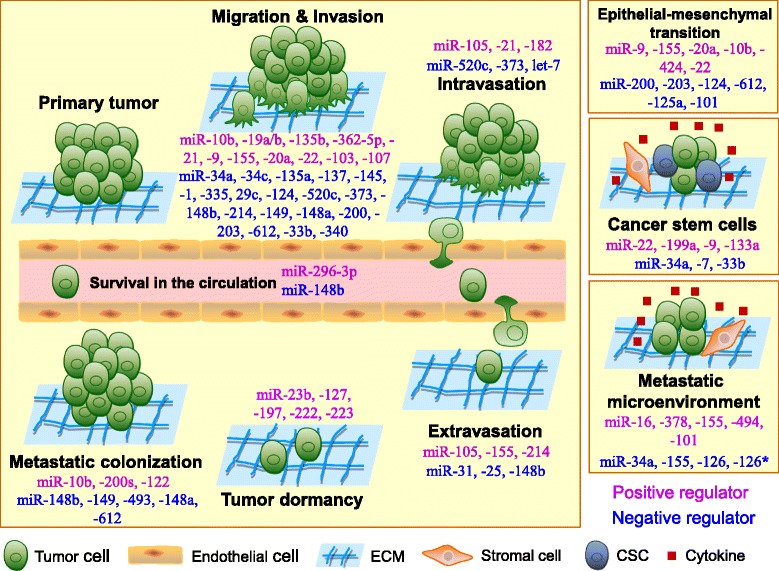
The roles of microRNAs in the regulation of tumor metastasis. Tumor metastasis is a complex process that is composed of multiple steps. To disseminate to distant sites, tumor cells detach from their primary sites by local migration, invasion and penetration of the stromal cell layers. For blood vessel-borne metastasis, disseminated tumor cells (DTCs) intravasate into blood vessels and survive in the circulation. After they arrive at distant organ sites, DTCs must extravasate from the blood and adapt to the new tissue microenvironments, where only a few DTCs form micrometastases. Finally, only a small subset of micrometastases eventually becomes detectable macrometastases. During this metastatic cascade, miRNAs can regulate the expression of multiple target genes and can modulate multiple tumor cell phenotypes, such as motility, invasion, intravasation, resistance to anoikis, extravasation and metastatic colonization as well as epithelial-mesenchymal transition, cell stemness, dormancy and the tumor microenvironment. miRNAs may act as positive regulators (purple) or negative regulators (blue) in the regulation of tumor metastasis. ECM, extracellular matrix; CSC, cancer stem cell

Current evidence has demonstrated that various miRNAs play critical roles in the coordination of tumor cell invasion, intravasation, survival, extravasation and/or colonization, as well as EMT, stemness, dormancy and the metastatic microenvironment (Fig. 1) (Table 1). Although metastasis-related miRNAs have opened a new field of research with respect to tumor metastasis, research on the functions and mechanisms of miRNAs in tumor metastasis has only recently begun.

**Table 1:** Functions of microRNAs in tumor metastasis

microRNA	Function in tumor metastasis	Targets and putative targets	References
miR-10b	Promotes cell migration, invasion and metastasis	TP53, PAX6, NOTCH1, HOXD10	[11, 12]
miR-19a/b	Promotes gastric cancer cell migration, invasion and metastasis	MXD1	[13]
miR-135b	Promotes cell migration, invasion and metastasis	LATS2, β-TrCP, NDR2, LZTS1	[14]
miR-362-5p	Promotes cell proliferation, migration and invasion *in vitro* and tumor growth and metastasis *in vivo*	CYLD	[15]
miR-34a	Inhibits cell migration, invasion and lung metastasis of breast cancer cells; suppresses prostate CSCs and metastasis; decreases the production of the chemokine CCL22; disturbs the bone metastatic niche	Fra-1, CD44, CCL22, Tgif2	[16, 68, 72, 73]
miR-34c	Inhibits cell migration, invasion and lung metastasis of breast cancer cells	Fra-1	[16]
miR-135a	Inhibits prostate cancer cell migration and invasion	ROCK1, ROCK2	[17]
miR-137	Reduces the invasiveness of colorectal cancer cells	FMNL2	[18]
miR-145	Attenuates gastric cancer cell migratory and invasive abilities *in vitro* and suppresses the metastatic cascade *in vivo*; inhibits the invasion and metastasis of neuroblastoma cells	N-cadherin, HIF-2α	[19, 20]
miR-1	Affects the cellular organization of F-actin and impairs tumor cell invasion and filopodia formation	FN1, LASP1, XPO6	[21]
miR-335	Suppresses cell migration, invasion and metastasis	Tenascin C, SOX4	[24]
miR-29c	Inhibits tumor invasion and metastasis	Collagens, Laminin γ1	[25]
miR-21	Promotes epithelial collective cell migration, invasion and lung metastasis; enhances colorectal cancer cell intravasation	TPM1, PDCD4, Maspin, PDCD4	[27, 30, 31]
miR-124	Modulates the intercellular adhesion of leading cells; inhibits EMT *in vitro* and suppresses intrahepatic and pulmonary metastasis *in vivo*	Integrin β1, ROCK2, EZH2	[28, 55]
miR-105	Destroys the integrity of vascular endothelial barriers to promote metastasis	ZO-1	[29]
miR-182	Promotes cancer cell intravasation into the circulation	Mtss1, Pai1, Rsu1, Timp1	[32]
miR-520c/373	Inhibits cell invasion *in vitro* and the intravasation of breast cancer cells *in vivo*	RELA, TGFBR2	[33]
let-7	Inhibits bone metastasis of breast cancer cells	HMGA2, Snail	[34]
miR-296-3p	Promotes prostate cancer cell survival	ICAM-1	[35]
miR-148b	Inhibits multiple steps of tumor progression via the regulation of invasion, resistance to anoikis, extravasation, lung metastasis colonization and chemotherapeutic response	ITGA5, ROCK1, PIK3CA/p110α, NRAS, CSF1	[36]
miR-155	Negatively regulates the function of the brain endothelial barrier; promotes TGF-β-induced EMT, tight junction dissolution, cell migration and invasion. Suppresses tumor growth via the regulation of the tumor microenvironment	Annexin-2, Claudin-1, DOCK-1, Syntenin-1, c-Maf, Pu.1, Ship1, C/EBPβ, HIF-1α	[38, 50, 76–78]
miR-214	Suppresses cell migration and invasion *in vitro* and inhibits liver metastasis of colorectal cancer cells *in vivo* but promotes migration, invasion and resistance to anoikis of melanoma cells *in vitro* and the extravasation and lung metastasis formation *in vivo*	FGFR1, TFAP2C	[39–41]
miR-31	Represses local invasion, extravasation and initial survival in distant tissues and distant metastatic colonization	ITGA5, RDX, RhoA	[42]
miR-25	Inhibits extravasation of prostate cancer cells *in vivo*	αv- and α6-integrins	[43]
miR-200s	Inhibits EMT but promotes metastatic colonization	Sec23a, ZEB1, ZEB2	[44, 52, 53]
miR-122	Promotes metastatic colonization of breast cancer cells	PKM2	[45]
miR-149	Inhibits basal-like breast cancer cell migration and invasion *in vitro* and impairs lung colonization *in vivo*	Rap1a, Rap1b	[46]
miR-493	Inhibits the settlement of metastasized colon cancer cells in the liver; promotes the death of colon cancer cells	IGF1R	[47]
miR-148a	Inhibits hepatoma cell migration *in vitro* and pulmonary metastatic colonization *in vivo*	Met	[48]
miR-9	Promotes breast cancer cell motility and invasiveness; enhances squamous cell carcinoma CSC expansion and metastasis	CDH1, α-catenin	[49, 66]
miR-20a	Induces EMT and promotes metastasis	Smad7	[51]
miR-203	Suppresses cell invasion and EMT *in vitro* and lung metastatic colonization *in vivo*	SNAI2	[54]
miR-612	Inhibits local invasion and distant metastatic colonization	Akt2	[56]
miR-125a	Inhibits EMT of ovarian cancer cells	ARID3B	[58]
miR-424	Promotes EMT	TGFBR3	[60]
miR-22	Promotes EMT, invasiveness, stemness and metastasis	TET1, TET2, TET3	[64]
miR-199a	Endows breast cancer cells with enhanced CSC properties; promotes invasion, angiogenesis and colonization in the case of melanoma	FOXP2, ApoE	[65, 92]
miR-133a	Promotes invasion of CSCs that express high levels of CD133	SGMS2, UBA2, SNX30, ANXA2	[67]
miR-7	Suppresses cell invasion and metastasis; inhibits the ability of breast CSCs to metastasize to the brain	SETDB1, KLF4	[69, 70]
miR-33b	Inhibits the stemness and lung metastasis of breast cancer cells	HMGA2, SALL4, Twist1	[71]
miR-494	Promotes the accumulation of MDSCs in tumor tissues	PTEN	[81]
miR-101	Augments cancer cell stemness and enhances the tumorigenic and metastatic potential of cancer cells; inhibits HCC growth, intrahepatic metastasis and distant metastasis	CtBP2, ROCK2	[82, 94]
miR-126	Inhibits endothelial recruitment, metastatic angiogenesis and metastatic colonization at distant sites	IGFBP2, PITPNC1, MERTK, SDF-1α	[83, 84]
miR-126*	Suppresses breast cancer metastasis via the inhibition of the recruitment of mesenchymal stem cells and inflammatory monocytes	SDF-1α	[84]
miR-23b	Promotes breast cancer cell dormancy in a metastatic niche	MARCKS	[87]
miR-127/197/222/223	Promotes breast cancer cell quiescence	CXCL12	[88]
miR-103/107	Promotes local invasion and liver metastasis	DAPK, KLF4	[89]

Interestingly, miRNAs in vesicles secreted from highly metastatic tumor cells can be internalized by weakly metastatic cells or stromal cells and subsequently influence the functions of these cells. Conversely, some miRNAs that are secreted from stromal cells can modulate the metastatic ability of DTCs. Thus, secretory miRNAs provide versatile communication between cancer cells and stromal cells and regulate the capacity for tumor growth at metastatic lesions. Moreover, the context-dependent roles of miRNAs in the metastatic cascade often involve multiple regulatory loops. The altered expression of one miRNA can regulate the expression of multiple target genes and modulate multiple cancer cell phenotypes, such as motility, invasion, resistance to anoikis and metastatic colonization. However, little is known about the roles of miRNAs in the regulation of DTC intravasation, circulation and extravasation. In addition, compared with conventional metastasis-related transcription factors, in most cases, miRNAs do not function as master regulators in the hierarchy of gene regulators. miRNAs may behave as critical tuners of transcriptional programs through the coordination of regulatory signaling networks that allow cells to adapt to microenvironmental and intracellular stresses during tumor metastasis. Comparisons of miRNA expression profiles of metastatic tumors in secondary sites with those of corresponding normal tissues and primary tumors have demonstrated that alterations in miRNA expression profiles can serve as phenotypic signatures of particular types of tumor metastasis and of specific steps in the tumor metastatic cascade. Therefore, miRNA expression profiling to screen for miRNAs that may serve as diagnostic or prognostic biomarkers or as therapeutic targets for tumor metastasis has great potential. However, much research is still needed for the development of effective miRNA-targeted therapies. A complete and accurate understanding of the molecular mechanisms of miRNAs in tumor metastasis will reveal invaluable diagnostic and prognostic biomarkers as well as potential targets for the therapeutic intervention of tumor metastasis.
